# Information

**Published:** 1888-08

**Authors:** 


### Information.

Dr. C. E. Dunn, Louisville, Ky., a member of the local committee of arrangements for the joint meeting of the American and Southern Dental Associations, gives the following information :

Hotel rates—The Louisville Hotel, $2.50 to $3.00 per day; Galt House, $2.50 to $4.00 per day—according to accommodations given ; Fifth Avenue Hotel, $1.75 per day ; Phœnix, $2.00 per day ; Alexander's, $2.00 per day; Rufer's Hotel (European), for rooms $1.00 per day.

Railroad rates—The "Southern Passenger Association," which controls territory south of the Ohio and east of the Mississippi rivers, one and one-third fare for the round trip ; the "Central Traffic Association" makes the same rate. The rate from both these associations is on the *certificate* plan. That is delegates should purchase regular ticket to Louisville, securing from the selling agent a certificate that they have purchased ticket by a stipulated route, which certificate when endorsed by the secretary of the association, and presented to the ticket agent of the road over which they arrived in Louisville, they will be sold tickets to the points from which they started for one-third rate—thus making a rate of one and one-third fare for the round trip. The "Chesapeake & Ohio Railroad" will sell from Washington City, Richmond, Charlottsville, Staunton, Va., and all points on said line, at one fare for the round trip. Tickets will be on sale August 24th, 25th and 26th, good to return until September 10th. The "Texas Traffic Association" say, "We have placed on sale, on all Lines in our association, summer excursion tickets to Louisville at one and one-third fare, and suggest that delegates to our convention avail themselves of same. "The Western States Passenger Association" have fixed the rate at one and one third for the round trip on the certificate plan, *asking* the Agent of whom they purchase their tickets for the certificate. The Trunk Line fix the rate at one and one third. Certificates to be had of Dr. Cushing, Secretary of the A. M. D. A. on application. These tickets are good until October 31st.

